# Uncertainty in action-value estimation affects both action choice and learning rate of the choice behaviors of rats

**DOI:** 10.1111/j.1460-9568.2012.08025.x

**Published:** 2012-04

**Authors:** Akihiro Funamizu, Makoto Ito, Kenji Doya, Ryohei Kanzaki, Hirokazu Takahashi

**Affiliations:** 1 JSPS Research FellowIchibancho 8, Chiyoda-ku, Tokyo 102-8472, Japan; 2 Neural Computation Unit, Okinawa Institute of Science and Technology1919-1 Tancha, Onna-son, Kunigami, Okinawa 904-0412, Japan; 3 Graduate School of Information Science and Technology, The University of TokyoHongo 7-3-1, Bunkyo-ku, Tokyo 113-8656, Japan; 4 Research Center for Advanced Science and Technology, The University of TokyoKomaba 4-6-1, Meguro-ku, Tokyo 153-8904, Japan; 5 PRESTO, JST4-1-8 Honcho Kawaguchi, Saitama 332-0012, Japan

**Keywords:** ambiguity, Bayesian inference, Bayesian Q-learning, neuroeconomics, reinforcement learning

## Abstract

The estimation of reward outcomes for action candidates is essential for decision making. In this study, we examined whether and how the uncertainty in reward outcome estimation affects the action choice and learning rate. We designed a choice task in which rats selected either the left-poking or right-poking hole and received a reward of a food pellet stochastically. The reward probabilities of the left and right holes were chosen from six settings (high, 100% vs. 66%; mid, 66% vs. 33%; low, 33% vs. 0% for the left vs. right holes, and the opposites) in every 20–549 trials. We used Bayesian Q-learning models to estimate the time course of the probability distribution of action values and tested if they better explain the behaviors of rats than standard Q-learning models that estimate only the mean of action values. Model comparison by cross-validation revealed that a Bayesian Q-learning model with an asymmetric update for reward and non-reward outcomes fit the choice time course of the rats best. In the action-choice equation of the Bayesian Q-learning model, the estimated coefficient for the variance of action value was positive, meaning that rats were uncertainty seeking. Further analysis of the Bayesian Q-learning model suggested that the uncertainty facilitated the effective learning rate. These results suggest that the rats consider uncertainty in action-value estimation and that they have an uncertainty-seeking action policy and uncertainty-dependent modulation of the effective learning rate.

## Introduction

The theory of standard reinforcement learning (Sutton & Barto, 1998), which mainly focuses on the reward expectation in the striatum and the reward prediction error in midbrain dopamine neurons, can predict the reward-estimation-based action selection of animals and humans (for review, see Daw & Doya, 2006; Corrado & Doya, 2007; O’Doherty *et al.*, 2007; Dayan & Niv, 2008). The theory only utilizes the expectation of reward estimation; however, in many cases, the estimation contains uncertainty (Rushworth & Behrens, 2008). Economists differentiate uncertainty from risk; uncertainty refers to the unknown reward-probability distribution, whereas risk refers to the variance of known reward-probability distribution (Epstein, 1999; Huettel *et al.*, 2006; Christopoulos *et al.*, 2009; Tobler *et al.*, 2009; O’Neill & Schultz, 2010). As most previous studies were based on the standard reinforcement learning theories, the neural substrate of uncertainty is unclear, and even worse, it is still elusive whether animals and humans consider uncertainty for action selection.

A possible role of uncertainty is in action choice; animals can have uncertainty-seeking or uncertainty-avoiding action policies. The concept of uncertainty seeking, or an exploration bonus, is included in reinforcement learning models (Dayan & Sejnowski, 1996; Dearden *et al.*, 1998; Daw *et al.*, 2006), although the models have not been verified with studies of animal choice behaviors. The theory of attention hypothesizes that uncertainty increases the salience of a cue (Esber & Haselgrove, 2011), and it may subsequently lead to facilitate action selection. In contrast, the concept of uncertainty avoiding, or an uncertainty aversion, is mainly proposed in economic theories derived from the observation of human behaviors (Epstein, 1999), often tested in a one-shot gambling task (e.g. Ellsberg, 1961). Thus, although various fields address uncertainty-dependent action choice, uncertainty dependence in animal choice behaviors is elusive.

Another potential role of uncertainty is the uncertainty-dependent time-varying learning rate (Daw *et al.*, 2006). In the Bayesian inference framework including the Kalman filter, after an observation of reward, the posterior distribution of reward shifts quickly when the previous distribution is flat, whereas it can shift less when the distribution is sharply peaked. It has been reported that the model with a time-varying learning rate captured the choice behaviors of animals and humans well (Behrens *et al.*, 2007; Ito & Doya, 2009). However, there is no direct evidence that the learning rate is affected by the reward uncertainty and neither has it been tested whether animal choice behaviors follow Bayesian uncertainty-dependent learning rate changes.

The aim of this study was to investigate the roles of uncertainty in action choice and learning by focusing on the uncertainty-dependent action modulation and time-varying learning rate. We tested whether Bayesian Q-learning models (Dearden *et al.*, 1998; Daw *et al.*, 2005) that keep track of the uncertainty of action values can fit the choice behaviors of rats better than standard Q-learning models that consider only the mean of the action values. The model analysis revealed that rats show an uncertainty-seeking action policy and use an uncertainty-dependent learning rate.

## Materials and methods

### Behavioral task

All procedures were approved by the institutional committee at the University of Tokyo and performed in accordance with the ‘Guiding Principles for the Care and Use of Animals in the Field of Physiological Science’ of the Japanese Physiological Society. We used five male Long-Evans rats (310–380 g each). Food was provided after the task to maintain the animals’ body weight at no less than 85% of the initial level. Water was supplied freely.

**Fig 1:**
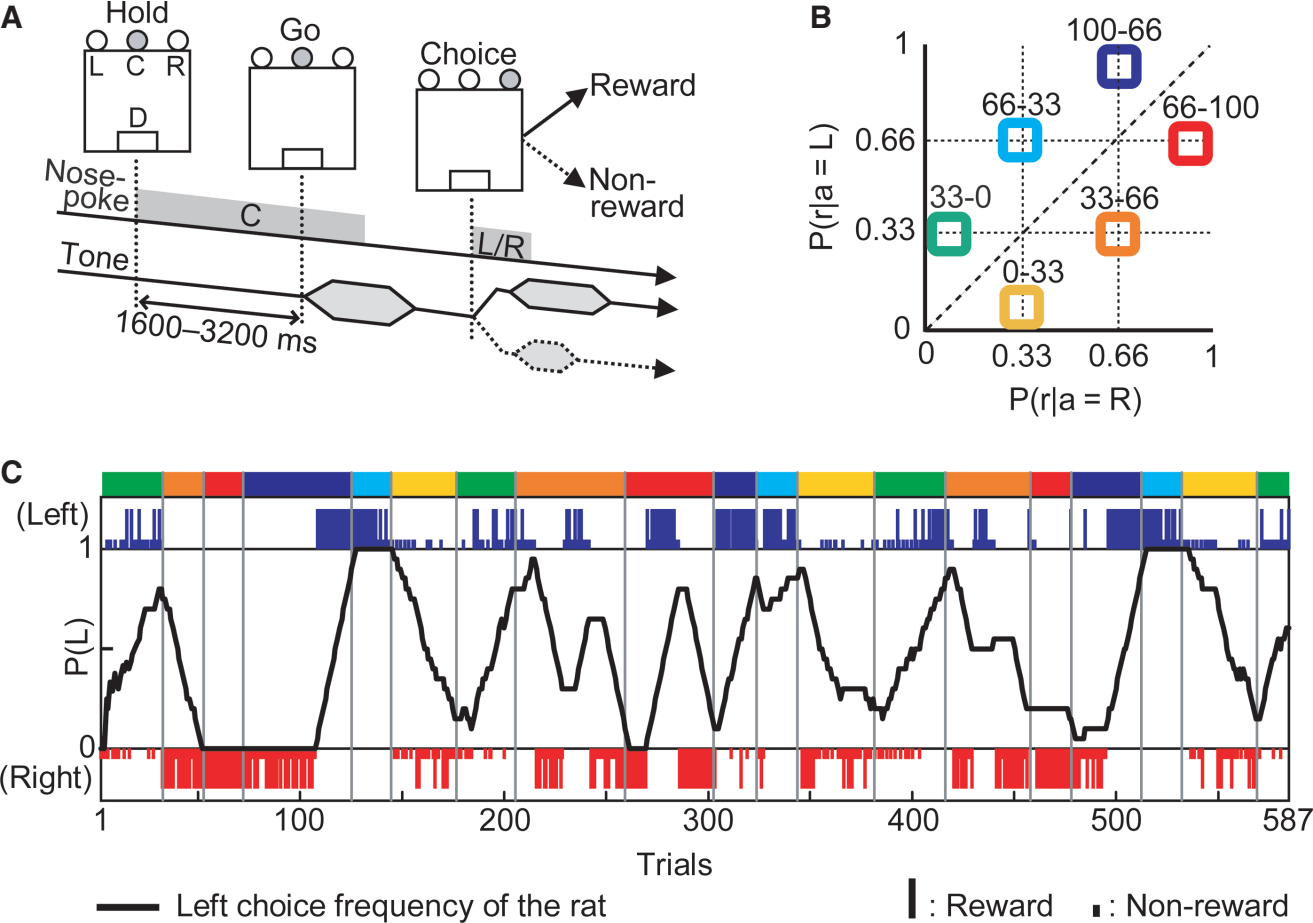
Free-choice task. (A) Task procedure. Rats first perform a nose-poke to the center hole [C], and they continue to poke for 1600–3200 ms until a Go tone is presented [Hold]. After the presentation of the Go tone [Go], the rats select either a left [L] or right [R] hole and receive a reward of a food pellet stochastically from a dispenser [D] [Choice]. (B) Reward-probability setting. We provided six reward-probability settings. The setting for the left and right holes changes when a rate of selecting a more rewarding hole of the rats in the last 20 trials reaches 80%. (C) Example of the choice behaviors of a rat. The vertical bars in the upper and lower portions of the figure indicate a left and right choice in each trial, respectively. The long and short bars show the reward and non-reward trials, respectively. The black line in the center indicates the left-choice frequency of the rat in the last 20 trials. The reward-probability setting of each block is shown by the color box on the top. The colors correspond to the reward-probability settings in B.

All experiments were conducted in a 36 × 36 × 37 cm experimental chamber (O’Hara & Co. Ltd) placed in a sound-attenuating box. The experimental chamber had three nose-poke holes on a wall and a pellet dish on the opposite side of the wall, as shown in Fig. 1A. All durations of poking, presence and consumption of the pellet were captured with infrared sensors and were recorded with a sampling rate of 1 kHz (Cerebus Data Acquisition System; Cyberkinetics Inc.).

Figure 1A shows our free-choice task. Rats first performed a nose-poke to the center hole, and they continued poking until a Go tone with a frequency of 5 kHz, an intensity of 50 dB sound pressure level (relative to 20 μPa) and a duration of 500 ms was presented (Hold). If the rats failed to continue poking, they were presented with an error tone (1 kHz, 70 dB sound pressure level, 50 ms), and the trial became an error. After the presentation of the Go tone, the rats selected either the left or right choice within 15 s and received a reward of a food pellet (25 mg) stochastically. A reward tone (20 kHz, 70 dB sound pressure level, 2000 ms) was presented immediately after the choice in a rewarded trial. In contrast, a non-reward tone (1 kHz, 70 dB sound pressure level, 50 ms) was presented in a non-rewarded trial. If rats did not select choices within 15 s from the presentation of the Go tone, the error tone was presented, as in an error trial.

The task consisted of six reward-probability settings (low, 33–0%; mid, 66–33%; high, 100–66%) for the left–right choices and the opposites, as shown in Fig. 1B. Among the settings, although the differences of reward probabilities were equal, the risk varied; the choices with the reward probabilities of 33 and 66% had higher risk values than did the choices with the reward probabilities of 0 and 100% (Fiorillo *et al.*, 2003; Rushworth & Behrens, 2008; Schultz *et al.*, 2008). Trials with the same reward-probability setting were referred to as a block, which consisted of at least 20 trials. Subsequently, the block changed when the rate of selecting the more rewarding hole reached 80% in the last 20 trials (Ito & Doya, 2009). The block change was conducted so as to: (i) include all of the six reward-probability settings in each of the six blocks and (ii) not repeat any of the settings. Each rat performed at least six blocks per day or per session, and any sessions consisting of fewer than seven blocks were not used in the analysis.

### Behavioral analysis

We investigated how well the choice behaviors of the rats fit to standard Q-learning models, which consider the expectation of reward but ignore the uncertainty, and Bayesian Q-learning models, which consider not only the expectation but also the probability distribution, including the uncertainty, of the reward for the action selection (Dearden *et al.*, 1998; Daw *et al.*, 2005). We tested whether the rats considered uncertainty during reward estimation and whether the rats utilized an uncertainty-dependent time-varying learning rate or a fixed learning rate. We also tested whether the uncertainty-dependent action modulation was essential for the choice behaviors of the rats by investigating how well the Bayesian Q-learning model with or without action modulation captured the behaviors. In the behavioral analyses, the error trials (in which the rats failed to continue poking to the center hole or took more than 15 s to select a left or right choice) were removed, and the remaining sequences of the success trials (in which the rats successfully selected the left or right choice) were used.

#### Standard Q-learning model

The standard Q-learning model updates the expectation of reward values for left and right choices (i.e. action values) with past actions and rewards, and predicts the choice probability (Watkins & Dayan, 1992; Sutton & Barto, 1998). In our task, the values of left and right choices were updated independently such that an optimal choice could not be found by tracking the value of only one choice. For example, when the reward probability was 33%, rats could not know that the chosen option had a higher reward probability than the other, because the reward-probability setting was 33–66% or 33–0%. We denoted the action as 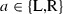 and the reward as 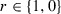, and we updated the action value in each trial, *Q*_*a*_(*t*), with the following equation (Ito & Doya, 2009):


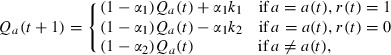
1

where *a*(*t*) and *r*(*t*) were the choice and reward at trial *t*, respectively. α_1_, α_2_, *k*_1_ and *k*_2_ were free parameters; α_1_ showed the learning rate in the chosen option, and α_2_ showed the forgetting rate in the unchosen option. Also, *k*_1_ and *k*_2_ indicated the strength of reinforcement in the reward and non-reward outcomes, respectively. The equation became an original Q-learning model when we set α_2_ = *k*_2_ = 0 (Sutton & Barto, 1998). Next, we referred to the model with α_1_ = α_2_ as the forgetting Q-learning (FQ-learning) model (Barraclough *et al.*, 2004), and we referred to the full four-parameter model as the differential FQ-learning model (Ito & Doya, 2009). A predicted choice probability was calculated with the following soft-max equation:


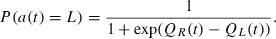
2

In each session, the free parameters were decided to maximize a normalized likelihood described later. The initial action values of the left and right choices were both 0.5 (i.e. the average reward probability of the six reward-probability settings in the task).

#### Bayesian Q-learning model

The Bayesian Q-learning model predicts not only the expectation but also the probability distribution, including the uncertainty, of the reward estimation for action selection. In this model, we assumed that: (i) the distribution of the action value, *Q*_*a*_, was expressed at each step as a beta distribution, *Beta*(*x*_*a*_,*y*_*a*_), to represent a binary random variable of the reward in this task (Daw *et al.*, 2005; Bishop, 2006); (ii) the distribution of *Q*_*a*_ was independent for each action candidate; and (iii) the distribution of *Q*_*a*_ changed in each time step to model a temporally changing environment. The action value, *Q*_*a*_, took a *q* ranging between 0 and 1, and the distribution could be obtained with Bayes’ theorem:


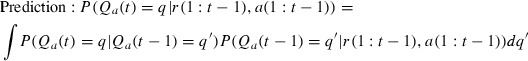
3


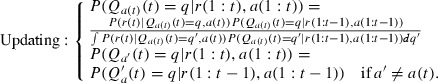
4

Equation 3 predicted the distribution of *Q*_*a*_ at trial *t* from the distribution in the previous trial. Equation 4 updated the distribution at trial *t* with the action and reward information at trial *t* when *a* = *a*(*t*), and this equation maintained the predicted distribution of Eqn 3 when *a* ≠ *a*(*t*). The transition probability in Eqn 3 controlled the change in the distribution variance in each trial, which was similar to setting the forgetting rate in a standard Q-learning model (Barraclough *et al.*, 2004). The transition probability was defined as follows:


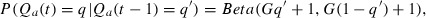
5

where *G* was a free parameter. In the transition probability, the beta distribution of *Q*_*a*_ at trial *t* retained the mode value of *Q*_*a*_ at trial *t*−1. When *G* had a small value, the variance of the distribution increased greatly at every time step. Although the integral in Eqn 3 was neither solvable nor a beta distribution, its mean and variance were analytical. Thus, we approximated *P*(*Q*_*a*_(*t*) = *q*|*r*(1:*t* − 1), *a*(1:*t* − 1)) in Eqn 3 as the beta distribution matching this average and variance:


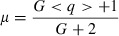
6


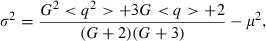
7

where <*q*> and <*q*^2^> were the first and second moments, respectively, of the beta distribution, 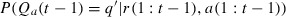. Based on the average and variance values observed in Eqns 6 and 7, the hyperparameters of the beta distribution, 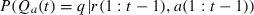, were subsequently obtained analytically by solving the following equations when the beta distribution was *Beta*(*x*,*y*):


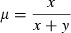
8


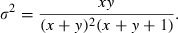
9

When the distribution *P*(*Q*_*a*(*t*)_(*t*) = *q*|*r*(1:*t* - 1), *a*(1:*t* - 1)) was *Beta*(*x*,*y*), Eqn 4 updated the distribution, *P*(*Q*_*a*(*t*)_(*t*) = *q*|*r*(1:*t*), *a*(1:*t*)), as follows:


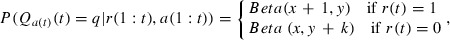
10

where *k* was a free parameter. In this equation, *k* indicated the relative strength of the reinforcement of the non-reward outcomes compared with the reward outcomes to model that the animals received reward and non-reward outcomes as the different magnitudes of reinforcers (Barraclough *et al.*, 2004; Ito & Doya, 2009). However, in a strict Bayesian process, *k* = 1; we referred to the model in which *k* = 1 as the original Bayesian Q-learning model. When *k* = 0, the Bayesian Q-learning model only utilized the reward outcomes; this model was referred to as the asymmetric Bayesian Q-learning (asymmetric BQ-learning) model. Next, we referred to the model with full-free parameters as the generalized Bayesian Q-learning (generalized BQ-leaning) model. Although the Bayesian Q-learning models did not use the exact learning rate, the effective learning rate, *e*α(*t*), was obtained analytically with an equation similar to that used to derive the learning rate, α_1_, in the standard Q-learning models:


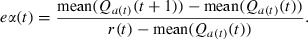
11

When the action-value distribution, *Beta*(*x*_*a*(*t*)_,*y*_*a*(*t*)_), was sharply peaked, the mean of the distribution slightly changed by a reward or non-reward event in each trial in the Bayesian inference framework, indicating a low effective learning rate. In contrast, a flat distribution led to a high effective learning rate.

In the Bayesian Q-learning models, the weighted sum of the mean and standard deviation of *Q*_*a*_ was utilized for action choice, and the prediction of the left choice probability was given by the following soft-max equation:


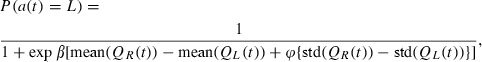
12

where β and ϕ were free parameters. When ϕ had a positive value, an option with a larger standard deviation was more likely to be selected, which was equivalent to the uncertainty-seeking or exploration bonus (Dayan & Sejnowski, 1996; Dearden *et al.*, 1998; Daw *et al.*, 2006). In contrast, when ϕ had a negative value, an option with a small standard deviation was selected (i.e. uncertainty aversion) (Epstein, 1999). In addition, when we set ϕ = 0, the model did not consider the uncertainty-dependent action modulation of the animals. Thus, ϕ served to test the effect of uncertainty-dependent action choice.

All of the free parameters of the Bayesian Q-learning models were determined such that the normalized likelihood was maximized. We set the initial values of the distribution of *Q*_*a*_ as *Beta*(1,1) in both the left and right choices in which the average *Q*_*a*_ was 0.5.

#### Model comparison

We employed the normalized likelihood to investigate how well the standard Q-learning and Bayesian Q-learning models fit the choice behaviors of the rats (Ito & Doya, 2009). The normalized likelihood, *Z*, was defined as follows:


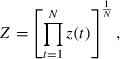
13

where *N* and *z*(*t*) were the number of trials and the likelihood at trial *t*, respectively. With the predicted left choice probability, *P*(*a*(*t*) = *L*), the likelihood, *z*(*t*), was defined as follows:


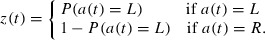
14

We conducted a 2-fold cross-validation for the model comparison. In the cross-validation, all of the sessions analysed were equally divided into two groups. One group provided the training data, and the other group provided the validation data. The free parameters of each model were determined such that the normalized likelihood of the training data was maximized. With the determined parameters, the normalized likelihood of each session in the validation data was analysed. We then switched the roles of the two groups of datasets and repeated the same procedure to obtain the normalized likelihoods in all sessions. The cross-validation analysis implicitly took into account the penalty of the number of free parameters (Bishop, 2006).

#### Role of uncertainty in choice behavior

In addition to the comparison of the normalized likelihood in the standard Q-learning and Bayesian Q-learning models, we further tested the roles of uncertainty in the action modulation and learning rate. We first analysed the free parameter of Bayesian Q-learning (i.e. ϕ, which was set to maximize the normalized likelihood in each session) to further probe the uncertainty-dependent action choice of the rats.

Next, we verified whether the learning rate changed on a trial-by-trial basis during the task; in the choice behaviors of the rats in the first and last 10 trials in each of the blocks, we independently fit a standard Q-learning model and identified the learning rate that achieved the highest normalized likelihood. The other free parameters were kept constant in each block to prevent any potential bias from the parameters. We also identified the effective learning rates of a Bayesian Q-learning model in the first and last parts of the blocks. Unlike the analysis of standard Q-learning, the analysis of Bayesian Q-learning employed exactly the same free parameters in the first and last parts of each block. Next, we tested whether the difference in the learning rates in the standard Q-learning between the two conditions was similar to that of the effective learning rate in the Bayesian Q-learning.

We elucidated the basic property of the effective learning rate in the Bayesian Q-learning models. We investigated the correlation between the effective learning rates and the mean or standard deviations of action values to verify whether the effective learning rate depended on uncertainty. We also employed a multiple regression analysis to further test the dependency of learning rate (Ito & Doya, 2009). The multiple regression analysis applied the following regression model to the effective learning rate, *e*α(*t*)


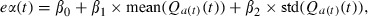
15

where *β*_i_ was the regression coefficient. When the effective learning rates correlated with the standard deviations of the action values, the model had a significant regression coefficient to the standard deviation (*t*-test, *P* < 0.01).

## Results

### Model-free behavioral analysis

Figure 1C shows an example of the choice behavior of a rat. The rat succeeded in changing its behaviors depending on the reward probabilities of the left and right choices. In this study, we analysed 130 sessions of data (rat 1, 31 sessions; rat 2, 48 sessions; rat 3, 27 sessions; rat 4, 12 sessions; rat 5, 12 sessions). The rats underwent 37.3 ± 0.578 trials (mean ± standard error, here and hereafter) for each block, and they experienced an average of 14.3 ± 1.71 blocks for each session.

**Fig 2:**
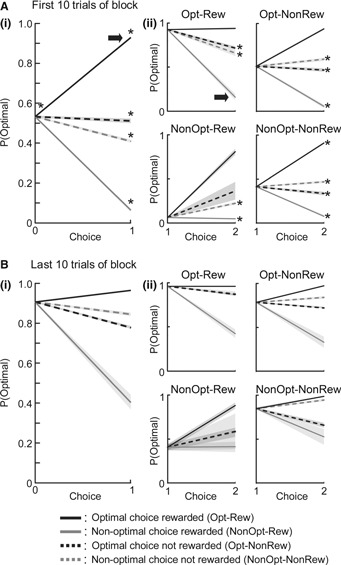
Experience-dependent change of optimal choice probability. The conditional probability of making a high reward probability choice (i.e. an optimal choice) is shown in the first (A) and last (B) 10 trials of each block, given the one (i) or two (ii) preceding experiences of the action and reward pairs. There are four types of experiences in each trial: optimal choice rewarded (Opt-Rew); non-optimal choice rewarded (NonOpt-Rew); optimal choice not rewarded (Opt-NonRew); and non-optimal choice not rewarded (NonOpt-NonRew). (i) The probability of selecting an optimal choice (Choice 0) and the conditional probability given the experience of the last trial (Choice 1). The mean and standard errors of the choice probabilities are shown by the lines and surrounding shaded areas, respectively. For example, the arrow in A(i) shows the conditional probability of selecting an optimal choice after experiencing Opt-Rew in the previous trial (92.9%). (ii) The conditional probability given the experiences of the last two trials are categorized by the experience of the second last trial shown at the top of each column. Each column shows the conditional probability of selecting an optimal choice given the experience shown at the top (Choice 1) and the conditional probability given the experiences of two previous trials (Choice 2). For example, the arrow in A(ii) shows the conditional probability of selecting an optimal choice after experiencing Opt-Rew and NonOpt-Rew in the second last trial, shown at the top, and the last trial, shown as the line, respectively (15.8%). The choice probabilities of all 130 sessions were compared between the first and last parts of the blocks (*P < 0.0001, Mann–Whitney *U*-test).

Figure 2 shows the conditional probability of making an optimal choice in the first (A) and last (B) 10 trials of each block given the experiences in one (i) or two (ii) preceding trials. There are four possible types of experiences in each trial: optimal choice rewarded; non-optimal choice rewarded; optimal choice not rewarded; and non-optimal choice not rewarded. For example, the arrow in Fig. 2A(i) shows the conditional probability of selecting an optimal choice after experiencing the optimal choice rewarded in the previous trial. The arrow in Fig. 2A(ii) shows the conditional probability after experiencing the optimal choice rewarded and non-optimal choice rewarded in the second last and last trial, respectively. Between the first and last parts of the blocks, the optimal choice probability was significantly different at choice 0 and choice 1 following all four types of experiences, as shown in Figs 2A(i) and B(i) (Mann–Whitney *U*-test, *P* = 3.79E-44–1.87E-6). The experiences of the last two trials were significantly differently affected in 11 out of 16 conditions as shown in Fig. 2(ii) (Mann–Whitney *U*-test, *P* = 3.28E-36–1.32E-4). These results indicate that the choice behaviors of rats were different between the first and last parts of the block even for the same experiences of the recent trials, suggesting that different learning rates were employed under the two conditions.

### Model-based behavioral analysis

#### Bayesian Q-learning

**Fig 3:**
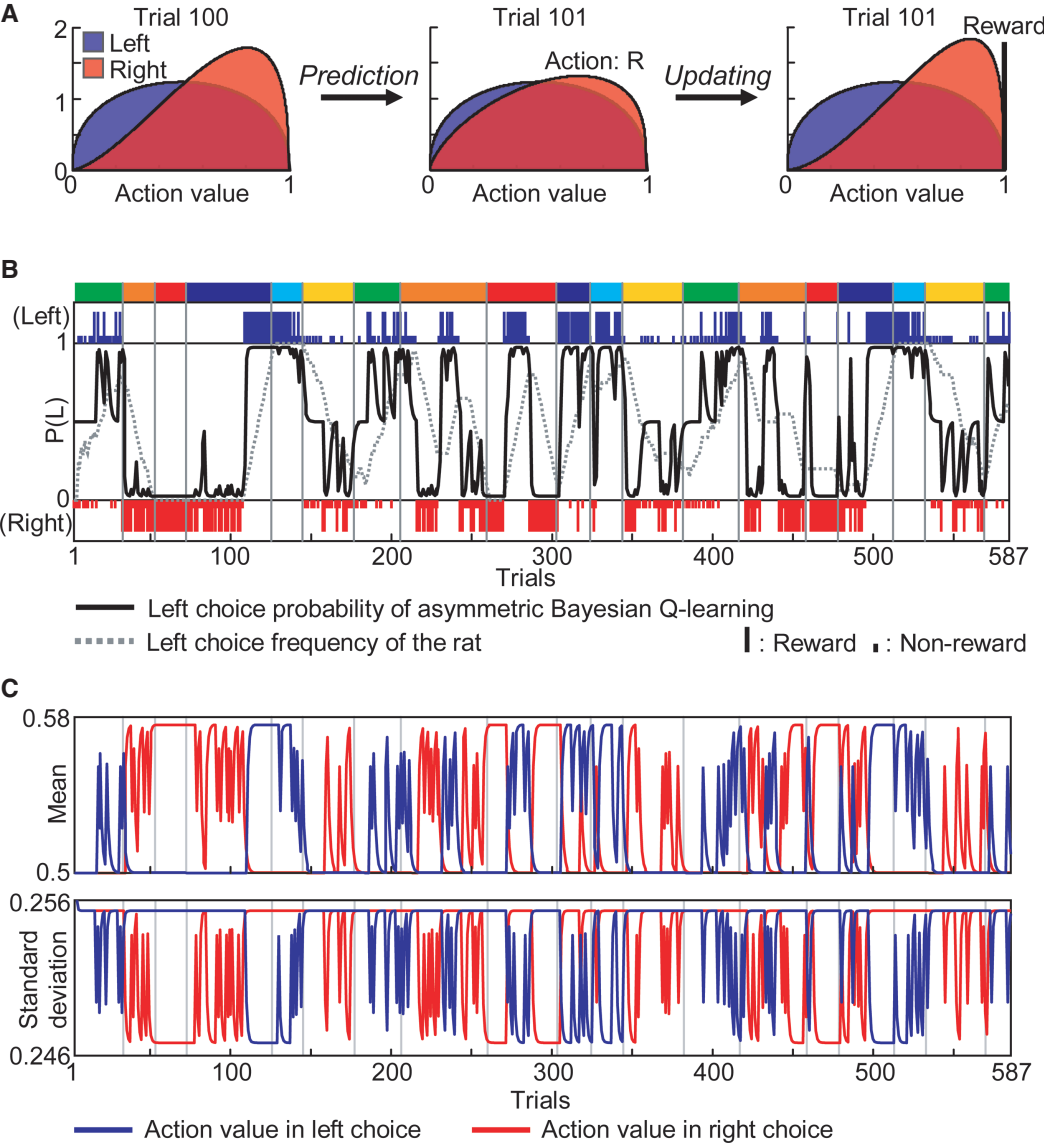
Bayesian Q-learning. (A) Procedure for updating the probability distribution of action value and computing the action selection probability. The probability distributions of action values in the asymmetric BQ-learning model for trials 100 and 101 in B are shown. In each time step, the distributions of both the left and right choices flatten with a forgetting effect (Prediction step). Based on the distributions, the action-selection probability of the rat is predicted. Next, the distribution of a chosen action is updated with the reward outcome, whereas that of the other action is maintained (Updating step). By repeating the procedure, the Bayesian Q-learning predicts the action selection probabilities in all trials. (B) Prediction of choice probability. The asymmetric BQ-learning predicted the choice probabilities of the behaviors of the rat in Fig. 1C. The free parameters of asymmetric BQ-learning were set to maximize the normalized likelihood in this session. The bold and dotted lines show the predicted left choice probability with the asymmetric BQ-learning and the choice frequency of the rat in the last 20 trials, respectively. The colors of boxes at the top correspond to the reward-probability settings in Fig. 1B. (C) Means and standard deviations of the probability distributions of action values. The means and standard deviations are shown in the upper and lower panels, respectively. The blue and red lines show the action values in the left and right choices, respectively.

Figure 3A shows the procedure for updating the probability distributions of the action values and computing the action selection probability by the Bayesian Q-learning model (see Materials and methods). Before a new trial starts, the action-value distributions from the previous trial flatten, corresponding to the forgetting or prediction of a possible environmental change (Prediction step). Based on both the mean and standard deviations of the distributions, either the left or right action was taken (Eqn 12). After the choice, depending on the reward outcome, the action-value distribution of the chosen action was updated, whereas that of the other action was maintained (Updating step). We predicted the choice behaviors of the rats in all of the trials by repeating the procedure. Figure 3B shows an example of the predicted choice probabilities in the asymmetric BQ-learning. Figure 3C shows the mean and standard deviation of the estimated action-value distribution. In trials immediately before the block change, the mean of the action value for the optimal choice became high. In contrast, the standard deviation of the action value tended to become low in the optimal action and tended to become high in the non-optimal, rarely chosen action. In this example, the mean action value was bounded to a certain ceiling around 0.58, because of the forgetting effect of the Bayesian Q-learning (Eqn 3).

#### Model comparison

**Fig 4:**
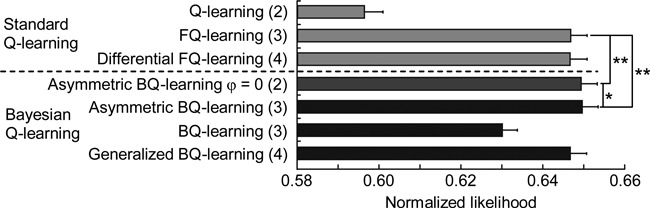
Normalized likelihoods of standard Q-learning and Bayesian Q-learning models. The results of the 2-fold cross-validation are shown. The normalized likelihoods of 130 sessions are compared among models. The mean and standard errors are presented. The number of free parameters in each model is shown in parentheses. *P < 0.05; **P < 0.01, paired *t*-test.

Figure 4 compares the normalized likelihood by 2-fold cross-validation for standard Q-learning models and Bayesian Q-learning models. Among the standard Q-learning models, the FQ-learning exhibited the highest normalized likelihood. Among the Bayesian Q-learning models, the asymmetric BQ-learning exhibited the highest normalized likelihood. By comparing the normalized likelihoods of the FQ-learning and asymmetric BQ-learning, we found that the asymmetric BQ-learning had higher normalized likelihoods in 99 of 130 sessions, and had a significantly higher normalized likelihood (paired t-test with 129 degrees of freedom, *P* = 1.28E-18). This result suggests that the choices of the rats depended on not only the mean values but also the distributions of the action values. We next compared the normalized likelihood of asymmetric BQ-learning and that with the action-choice parameter ϕ = 0, which does not consider the uncertainty in action choice. The asymmetric BQ-learning with ϕ = 0 showed a lower normalized likelihood (paired *t*-test, *P* = 0.0255), suggesting that the action choices of rats are modulated by uncertainty in action values. In addition, the asymmetric BQ-learning with ϕ = 0 showed a significantly higher normalized likelihood than the FQ-learning with a fixed learning rate (paired *t*-test, *P* = 1.03E-24), suggesting that not only the action choice but also the learning process is modulated by uncertainty.

#### Role of uncertainty in action choice

**Fig 5:**
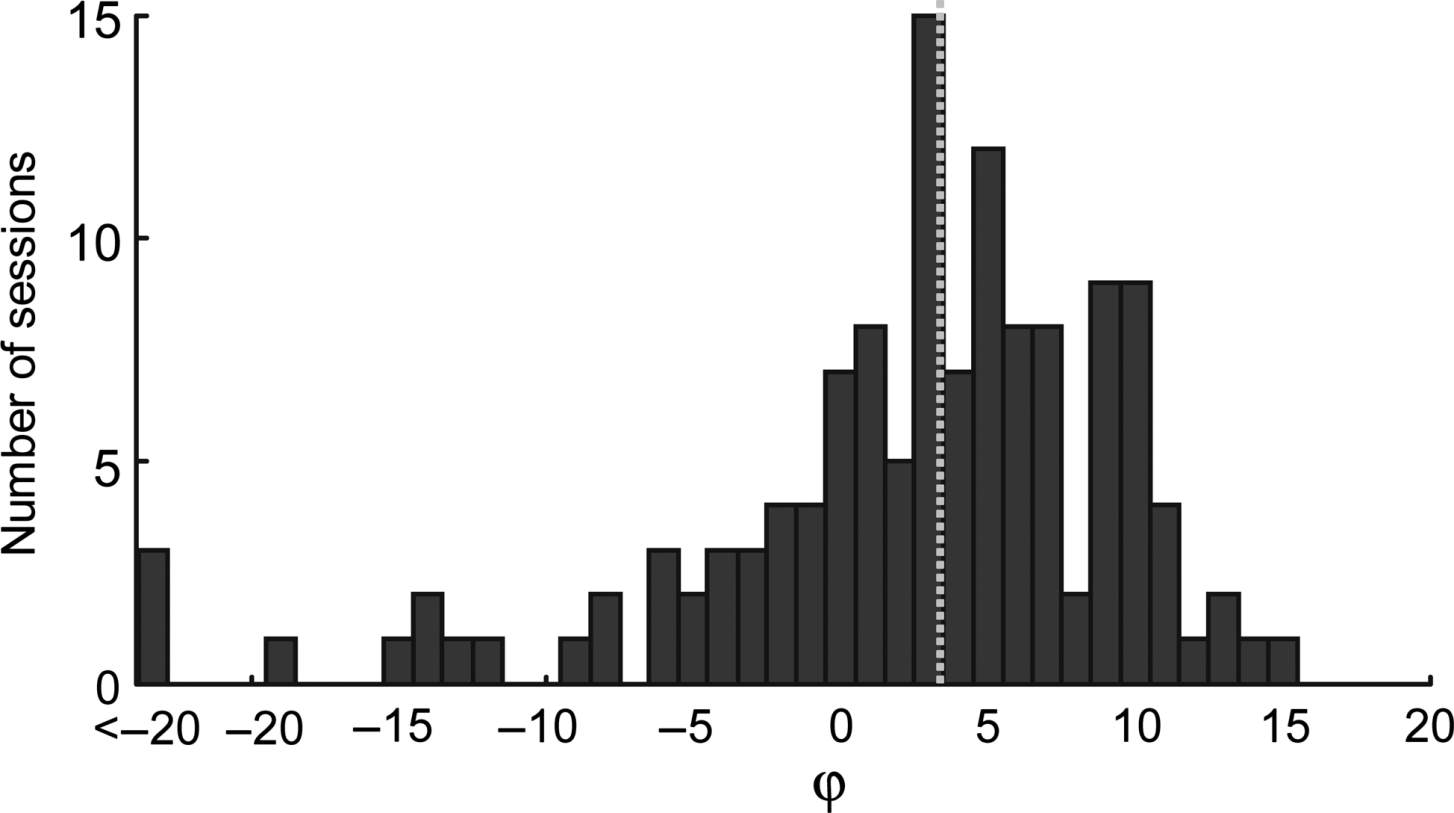
Uncertainty-dependent action modulation. Histogram of the free parameter, ϕ, in the asymmetric BQ-learning model. The free parameter was set such that the normalized likelihood of each session was maximized. The vertical dotted line shows the median value of ϕ (i.e. 3.47), which was significantly positive (Mann–Whitney *U*-test, *P* = 1.70E-6).

In order to assess the uncertainty dependence of the choice of the rats, we analysed the coefficient *ϕ* for the standard deviation of the action value in the action-choice equation (Eqn 12) of the asymmetric BQ-learning model, which showed the best fit of the animal behaviors. Figure 5 shows the distribution of the coefficient ϕ estimated in each session. The coefficient was significantly positive (Mann–Whitney *U*-test, *P* = 1.70E-6), which indicated that the rats were uncertainty seeking in this task.

#### Role of uncertainty in learning rate

**Fig 6:**
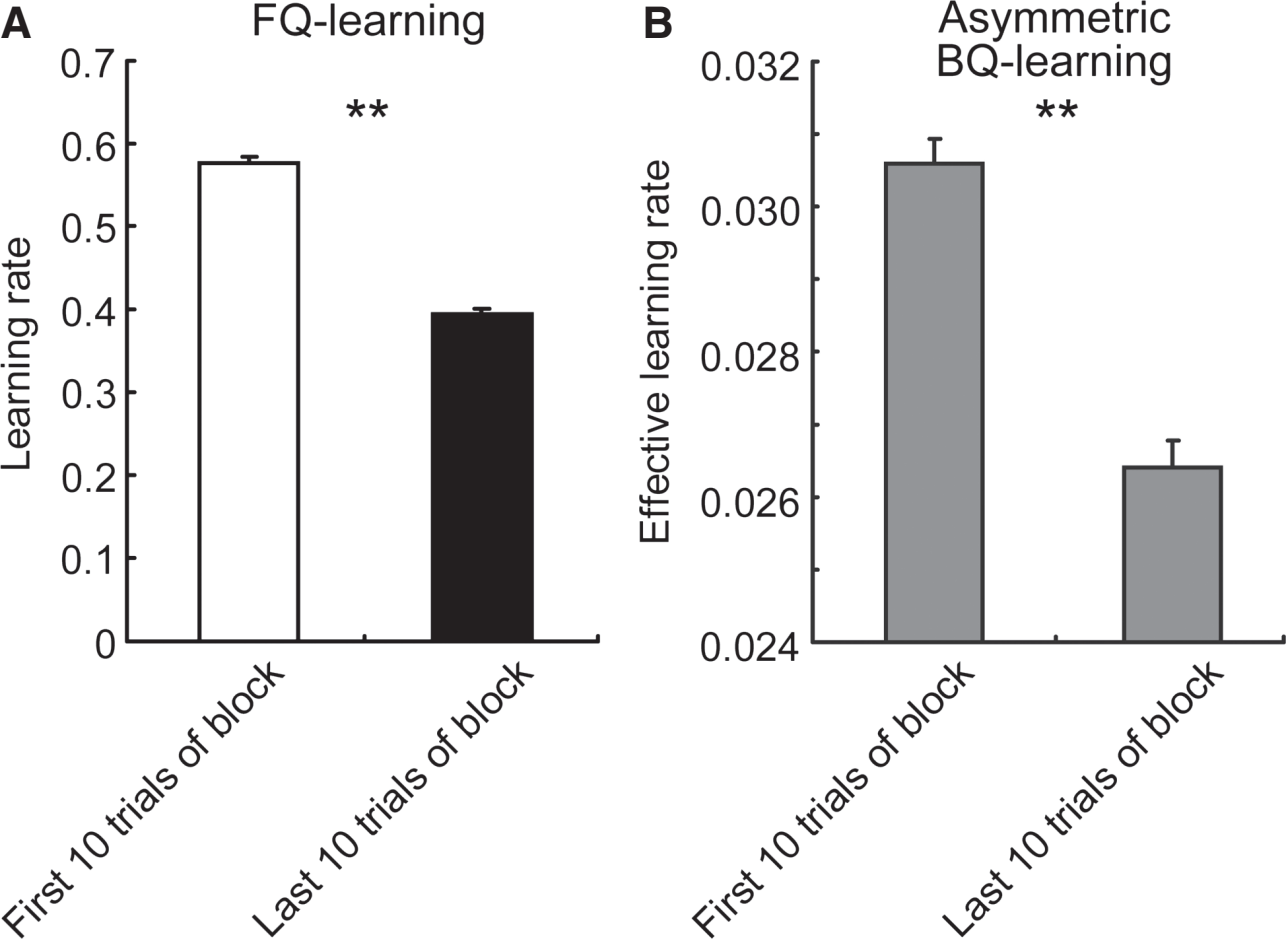
Time-varying learning rate. In the first and last 10 trials of each block, the learning rate (A) and effective learning rate (B) were investigated in the FQ-learning and asymmetric BQ-learning models, respectively. In the FQ-learning, the learning rates of the first and last 10 trials of each block were independently analysed to maximize the normalized likelihood. Thus, we could obtain the different learning rates for the two conditions. The other parameters were kept constant in each block. In contrast, in the asymmetric BQ-learning, the effective learning rates were analysed with the same free parameters in the first and last components of each block. 2630 blocks were analysed. The scales of the learning rate in A and B were different mainly because the FQ-learning and asymmetric BQ-learning employed different ranges of action values. The means and standard errors are shown (**P < 0.01, Mann–Whitney *U*-test).

Figure 6A shows the mean and standard errors of learning rates of FQ-learning in the first and last 10 trials of 2630 blocks. The learning rates of FQ-learning were independently set to achieve the highest normalized likelihood in each first and last part of a block. The learning rates associated with the first 10 trials were significantly higher than those of the last 10 trials (Mann–Whitney *U*-test, *P* = 8.12E-63), suggesting that rats utilized time-varying learning rates. In the Bayesian Q-learning models, the effective learning rate can vary with the uncertainty even if the same forgetting parameter *G* is used. Figure 6B shows the effective learning rate (Eqn 11) of asymmetric BQ-learning in the first and last 10 trials of each block with the same forgetting parameters. Similar to Fig. 6A, the effective learning rates of the first 10 trials were significantly higher than those of the last 10 trials (Mann–Whitney *U*-test, *P* = 2.79E-67). A possible reason for such differences in the effective learning rates in the first and last parts of the blocks is the modulation of learning rate by action-value uncertainty.

**Fig 7:**
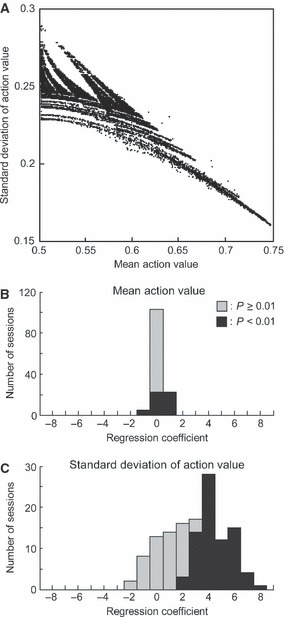
Uncertainty-dependent learning rate. (A) The relationship of the mean and standard deviations of action values estimated by the asymmetric BQ-learning. Each dot shows the mean/standard deviation pair of the distribution in each trial. The data for all 130 sessions are plotted. The free parameters of the asymmetric BQ-learning were set such that the normalized likelihood in each session was maximized. Regression coefficients of the means (B) and standard deviations (C) of action values for the effective learning rates (see Materials and methods). The means and standard deviations of action values in the asymmetric BQ-learning model were used, and the free parameters of the model were set such that the normalized likelihood in each session was maximized. The black or gray bar indicates a significant (*t*-test, *P* < 0.01) or non-significant (*P* ≥ 0.01) regression coefficient, respectively. The number of sessions that had significant coefficients in the means and standard deviations of action values was 49 and 77 out of 130, respectively.

Figure 7A plots the mean and standard deviation of the action value estimated by the asymmetric BQ-learning. Bayesian Q-learning could capture the average level and uncertainty of reward prediction separately in this task, although they were not independent. We analysed the correlation between the means or standard deviations of action values and the effective learning rates (see Materials and methods) in asymmetric BQ-learning. The average correlation coefficient between the mean action value and the effective learning rate was −0.175 ± 0.00827, and the correlations were significantly negative in 112 out of 130 sessions (*P* = 1.01E-33–0.0212). In contrast, the average correlation between the standard deviation of action value and the effective learning rate was 0.196 ± 0.00782, and the correlations were significantly positive in 119 out of 130 sessions (*P* = 4.91E-43–0.0472). Figures 7B and C show the regression coefficients of the means and standard deviations of action values, respectively, in the multiple regression analysis (see Materials and methods). The effective learning rates were significantly correlated with the standard deviations of action values in 77 of 130 sessions, whereas they were correlated with the means in only 49 sessions. These results suggest that the learning rates were modulated by uncertainty rather than by the average of action value (Wilcoxon signed-rank test, *P* = 7.50E-5).

## Discussion

To investigate the role of uncertainty in action choice and learning, we analysed choice behaviors in rats using a Bayesian Q-learning model that considered not only the expectation, but also the probability distribution of a reward for an action selection. First, a Bayesian Q-learning model that asymmetrically utilized reward and non-reward events (asymmetric BQ-learning) predicted the choice behaviors of the rats significantly better than standard Q-learning models that did not consider uncertainty in action choice and learning (Fig. 4). In addition, the asymmetric BQ-learning with uncertainty-dependent action modulation predicted the behaviors of the rats better than the model without it (Fig. 4). The analysis of the model parameter revealed uncertainty-seeking action modulation (i.e. an exploration bonus) for the rats in this task (Fig. 5). Second, the asymmetric BQ-learning without the uncertainty-dependent action modulation predicted the choice behaviors of rats better than standard Q-learning models (Fig. 4). Moreover, the effective learning rate of asymmetric BQ-learning was facilitated by uncertainty (Fig. 7C). These results suggest that the uncertainty-dependent time-varying learning rate was the reason for the significant difference in the action selections in the first and last stages of learning blocks (Figs 2 and 6).

### Bayesian Q-learning models

In the present and many other tasks with binary reward outcome, if the reward probability is *P*, the action value, or the expected reward, is *P* and the risk, or the variance of reward, is *P*(1−*P*) (Bishop, 2006; Preuschoff *et al.*, 2006, 2008; Rushworth & Behrens, 2008; Schultz *et al.*, 2008). The uncertainty in this study derives from the unknown reward probability *P* (Daw *et al.*, 2005, 2006). The learner estimates the action value *P* from repeated trials either by using its point estimate as in standard Q-learning, or by considering its probability distribution as in Bayesian Q-learning (Figs 3 and 7A). Note that another source of risk and uncertainty, the ambiguity of sensory input, is not addressed in this study (Knill & Pouget, 2004; Kepecs *et al.*, 2008; Kiani & Shadlen, 2009).

Although the various Bayesian Q-learning models can utilize the uncertainty-dependent action modulation and learning rate, a model should be selected to validate their pure effects. Ito & Doya (2009) recently proposed three varieties of standard Q-learning models to normatively explain animal choice behaviors. In keeping with previous observations regarding standard Q-learning models, we employed four varieties of Bayesian Q-learning models in this study (Fig. 4).

Our Bayesian Q-learning models assume that the variance of the distribution, or the uncertainty, increases in every time step, which is similar to setting the forgetting rate in the standard Q-learning models. This feature offers a better prediction of animal behaviors in the temporally changing environments, such as in a reversal task (Hampton *et al.*, 2006) or a free-choice task (Samejima *et al.*, 2005; Daw *et al.*, 2006; Ito & Doya, 2009), compared with the previous Bayesian Q-learning models that assume a stable environment (Dearden *et al.*, 1998; Daw *et al.*, 2005).

The asymmetric BQ-learning and generalized Bayesian Q-learning models utilize the asymmetric impact of reward and non-reward outcomes with the free parameter *k* (Eqn 10), in accordance with the recent findings that animals (Barraclough *et al.*, 2004; Ito & Doya, 2009) and humans (Kahneman & Tversky, 1979; Tversky & Kahneman, 1981; De Martino *et al.*, 2006) receive reward and non-reward outcomes with different magnitudes of reinforcers. However, when strictly considering a Bayesian process, *k* should equal 1 because a Bayesian process equally applies to each event, as in the original Bayesian Q-learning model. Thus, the free parameter, *k*, enables our Bayesian Q-learning models to explain normatively the animal choice behaviors by partly violating the Bayesian rule. The asymmetric BQ-learning demonstrated the highest normalized likelihood among the models (Fig. 4), thereby providing further support that our model captured the choice behaviors of the animals. The asymmetric BQ-learning with or without the uncertainty-dependent action modulation (i.e. ϕ = 0) served to distinguish the effects of uncertainty in both action choice and learning or in only learning.

In the Bayesian Q-learning framework, the posterior distribution of the action value from binary reward observation takes the beta distribution (Bishop, 2006), whereas it can in general be a Gaussian distribution or multimodal distributions for non-binary rewards. The beta distribution has only two degrees of freedom, which is why we focused on the mean and standard deviation of the action-value estimate. In order to analyse any effect of the higher order moments of the distribution (e.g. skewness) independently of the mean and standard deviation, a different task setting is required (Symmonds *et al.*, 2010, 2011).

Recent studies employ a model-based strategy and show that the strategy or a hybrid model of model-free and model-based strategy offers better prediction of human behaviors than only a standard model-free reinforcement learning model (Hampton *et al.*, 2006; Dayan & Niv, 2008; Glascher *et al.*, 2010). In this study, if the rats took a model-based strategy to predict the timing of block change, the learning rate could have been larger in the last part of the blocks. On the contrary, the estimated learning rates were lower in the last part of the blocks (Fig. 6). Thus, we inferred that the rats took a model-free strategy (i.e. standard Q-learning or Bayesian Q-learning model) and analysed the changes in the choice and learning rate in terms of the uncertainty of action values (Figs 5 and 7C).

### Role of uncertainty in choice

The model comparison and analysis of the free parameters in Bayesian Q-learning suggest that the uncertainty-dependent action choice (i.e. uncertainty seeking in this task) is essential for the choice behaviors of rats (Figs 4 and 5). This is consistent with the proposed reinforcement learning algorithms (Dayan & Sejnowski, 1996; Dearden *et al.*, 1998; Daw *et al.*, 2006). The uncertainty seeking, or exploration bonus, was originally proposed to balance exploration and exploitation, in which the agent encouraged the selection of long-ignored actions (Sutton, 1990; Dayan & Sejnowski, 1996). Our choice task might require such a behavior to find an optimal choice after a block change (e.g. the reward-probability setting changes from 66–33% to 66–100% in left–right choices).

### Role of uncertainty in learning

The asymmetric BQ-learning without the uncertainty-dependent action modulation provided better normalized likelihoods than did the standard Q-learning models, suggesting that the uncertainty-dependent learning rate is also essential for the choice behaviors (Fig. 4). One candidate role of uncertainty in learning is to change the learning rate temporally, as proposed in a recent study (Daw *et al.*, 2006). In our free-choice task, the action selections in the first and last parts of the blocks were significantly different (Fig. 2); the difference of action selection is possibly explained with the uncertainty-dependent time-varying learning rates in asymmetric BQ-learning (Fig. 6B). It is, in general, possible that the difference in the learning rate between the first and last parts of the blocks is affected by our task setting; a new block only began when the action selections of rats became stable (i.e. the rate of selecting the more rewarding hole reached 80%). Uncertainty was usually higher after the block change and became lower near the end of the block. However, the reward-probability settings (i.e. 0, 33, 66, and 100%) induced different levels of the risks and uncertainties even near the end of the blocks. Therefore, our task allowed us to investigate the effect of uncertainty separately from the effect of the number of experiences in the block.

### Hypothetical neural implementation of uncertainty

A recent study reports strong evidence that the activity of the anterior cingulate cortex (ACC) correlates with the volatility of the task environment (Behrens *et al.*, 2007). The study then suggests that the volatility induces uncertainty of reward estimation and changes the learning rates of humans (Rushworth & Behrens, 2008). This is consistent with our result; uncertainty affects the learning rate of rats. The ACC is also known to become active with a novel sensory stimulus rather than a familiar stimulus (Downar *et al.*, 2002; Gompf *et al.*, 2010), and the novelty should be related to uncertainty. The ACC projects to the locus coeruleus (LC), which is a major site of norepinephrine (NE) neurons (Aston-Jones & Cohen, 2005; Gompf *et al.*, 2010). The phasic and tonic activities of LC neurons seem to correspond to the exploitation and exploration behavior, respectively (Usher *et al.*, 1999), to modulate the action choice. In addition, many theoretical studies predict that NE controls the exploitation/exploration balance (Doya, 2002, 2008; Ishii *et al.*, 2002) or uncertainty (Yu & Dayan, 2005), inferring that the LC controls the uncertainty-dependent action choice. In addition to NE, acetylcholine (ACh) is thought to represent uncertainty (Yu & Dayan, 2002, 2005). ACh induces cortical plasticity (Froemke *et al.*, 2007; Blundon *et al.*, 2011) and associative learning (Letzkus *et al.*, 2011), suggesting that ACh controls the learning rate (Doya, 2002). ACh is delivered to the cortex from the basal forebrain, which also receives inputs from the ACC (Ongür *et al.*, 1998). Thus, the ACC/LC/basal forebrain possibly controls both the uncertainty-dependent action choice and learning rate.

Another possible mechanism for the coding of uncertainty is populational neural activities, in which the variance of neural activities serves to represent the uncertainty of reward prediction (Pouget *et al.*, 2003; Daw *et al.*, 2005). In keeping with the previous hypothesis, the population of action-value-representing neurons in the striatum (Doya, 2002, 2008; Samejima *et al.*, 2005; Pasquereau *et al.*, 2007; Lau & Glimcher, 2008) may encode uncertainty. Moreover, recent studies report that the activity of the orbitofrontal cortex represents sensory uncertainties (Hsu *et al.*, 2005; Kepecs *et al.*, 2008). The orbitofrontal cortex also projects to the LC and basal forebrain (Ongür *et al.*, 1998), potentially representing value uncertainty.

## Conclusion

Our study suggests that rats consider the uncertainty for action selection, and that the uncertainty-dependent action choice and learning are both essential for choice behaviors. Candidate brain areas for encoding uncertainty are the ACC, orbitofrontal cortex and striatum. In addition, recent studies have proposed the coding of uncertainty by neuromodulators, which suggests that ACh and norepinephrine levels reflect uncertainty. Thus, combining our Bayesian Q-learning models with the electrophysiological recording of the candidate brain areas and/or the neuromodulator measurements during the task leads to further understanding of the neural substrates of uncertainty.

## Abbreviations

ACC: anterior cingulate cortex
ACh: acetylcholine
asymmetric BQ-learning: asymmetric Bayesian Q-learning
FQ-learning: forgetting Q-learning
LC: locus coeruleus
